# Mercury Exposure in Birds of Prey from Norway: Relation to Stable Carbon and Nitrogen Isotope Signatures in Body Feathers

**DOI:** 10.1007/s00128-023-03740-6

**Published:** 2023-06-02

**Authors:** Pilar Gómez-Ramírez, Jan Ove Bustnes, Igor Eulaers, Trond Vidar Johnsen, Giles Lepoint, Juan Manuel Pérez-García, Antonio Juan García-Fernández, Silvia Espín, Veerle Leontina Bernard Jaspers

**Affiliations:** 1 Toxicology Group, Department of Health Sciences, Faculty of Veterinary, University of Murcia, Campus de Espinardo, 30100 Murcia, Spain; 2 Norwegian Institute for Nature Research, Fram Centre, 9296 Tromsø, Norway; 3 Department of Bioscience, Faculty of Technical Sciences, Aarhus University, Frederiksborgvej 399, PO Box 358, 4000 Roskilde, Denmark; 4 Laboratory of Oceanology, UR FOCUS, University of Liège, 4000 Liège, Belgium; 5 Ecology Area, Department of Applied Biology, University Miguel Hernández, 03202 Elche, Spain; 6 Environmental Toxicology Group, Department of Biology, Norwegian University of Science and Technology (NTNU), 7024 Trondheim, Norway

**Keywords:** Biomonitoring, Feather, Erythrocytes, Red blood cells, Metals

## Abstract

Mercury (Hg) and stable carbon and nitrogen isotope ratios were analysed in body feathers from nestlings of white-tailed eagles (*Haliaeetus albicilla)* (WTE; n = 13) and Northern goshawks (*Accipiter gentilis*) (NG; n = 8) and in red blood cells (RBC) from NG (n = 11) from Norway. According to linear mixed model, *species* factor was significant in explaining the Hg concentration in feathers (LMM;* p* < 0.001, estimate (WTE) = 2.51, 95% CI = 1.26, 3.76), with concentrations higher in WTE (3.01 ± 1.34 µg g^−1^ dry weight) than in NG (0.51 ± 0.34 µg g^−1^ dry weight). This difference and the isotopic patterns for each species, likely reflect their diet, as WTE predominantly feed on a marine and higher trophic-chain diet compared to the terrestrial NG. In addition, Hg concentrations in RBCs of NG nestlings were positively correlated with feather Hg concentrations (*Rho* = 0.77, *p* = 0.03), supporting the potential usefulness of nestling body feathers to biomonitor and estimate Hg exposure. Hg levels in both species were generally below the commonly applied toxicity threshold of 5 µg g^−1^ in feathers, although exceeded in two WTE (6.08 and 5.19 µg g^−1^ dry weight).

Mercury (Hg) is considered a global contaminant and a threat to human and environmental health, which has led to implementation of legislative measures to reduce its emissions at global and local scale (Climate and Pollution Agency 2010; UNEP 2013, 2021). The effectiveness of such restrictions and the current exposure concentrations in the environment can be assessed by biomonitoring using birds of prey (Gómez-Ramírez et al. 2014). In this sense, feathers are considered a good tool because most of the total Hg body burden (typically 70%–90%) is remobilised and sequestered into the growing feathers (Furness et al. 1986; Espín et al. 2016) which themselves reflect dietary Hg in chicks (Rattner et al. 2008; Lodenius and Solonen 2013; Ekblad et al. 2021). In the case of nestlings of territorial and non-migratory species, the information about local contamination status is even more accurate, as these are usually fed with prey caught close to the nest (Frank and Lutz 1999). While feathers integrate exposure during their growth, blood provides a non-destructive measure of recent contaminant exposure (Espín et al. 2014a, 2016).

To study dietary habits, carbon (C) and nitrogen (N) stable isotopes (SIs) are often considered as a better time-integrated approach than conventional use of pellets, regurgitates or stomach contents (Inger and Bearhop 2008). Moreover, when chick feathers are used, there is no or little temporal mismatch between SIs and Hg (Bond 2010; Carravieri et al. 2014).While *δ*^13^C is used to identify sources of C (i.e. marine, terrestrial, etc.), *δ*^15^N is very useful to assess trophic position in dietary studies and bioaccumulation of contaminants (Inger and Bearhop 2008). This approach has also been used in the field of ecotoxicology to investigate sources of pollutants (Ramos and González-Solís 2012; Eulaers et al. 2013, 2014; Gómez-Ramírez et al. 2017; Badry et al. 2019).

Our objective was to compare the exposure to Hg in nestling white-tailed eagles (WTE) (*Haliaeetus albicilla*), a marine top predator, and nestling Northern goshawks (NG) (*Accipiter gentilis*), a terrestrial bird-eating predator. Both species are resident in Northern Norway and have been part of ongoing biomonitoring of which this study is part. In addition, we aimed to relate Hg exposure to diet as proxied by stable C (*δ*^13^C) and N isotopes (*δ*^15^N) in body feathers and discuss the associated toxicological risk.

## Materials and methods

Samples were obtained in 2014 from two locations in Northern Norway (Nordland county: N 68.30–68.47°, E 24.54–25.27° and Troms and Finnmark county: N 68.77–67.39°, E 20.39–23.34° respectively). To our knowledge, there are no significant local sources of Hg pollution in the area (Dolan et al. 2017). When fledglings were at similar stages of feather development (NG were 18–26 days old and WTE were 35–56 days old (methods described in Gómez-Ramírez et al. (2017)), about 10 feathers from chest and back of each bird were gently plucked and kept in plastic bags at room temperature until analysis. At the same time, blood samples (1–4 mL) from NG were obtained by puncturing the brachial vein with a 23G needle and a heparin-coated syringe. Samples were transported refrigerated to the laboratory, where they were centrifuged at 5000 g for 10 min and plasma and RBCs were transferred into separate tubes and frozen at − 20°C until analysis (Espín et al. 2021). Sampling was approved by the Norwegian Food Safety Authority (Mattilsynet; 2014/58808-2).

Total Hg (henceforth Hg) was analysed in feathers from 13 WTE (from 9 nests) and 8 NG (from 3 nests) and RBCs from 11 NG (from 4 nests) at the Laboratory of Toxicology, University of Murcia (Spain) using a Milestone DMA-80 direct Hg analyser, by combustion-amalgamation atomic absorption spectrophotometry. Mercury in RBC and feathers of NG was measured in the same individuals, but due to the lack of samples, feathers of some nestlings could not be analysed. Following the method described by Espín et al. (2014a), one body feather (0.02–0.08 g dry weight; dw) was washed twice with distilled water, dried at room temperature, cut with scissors and loaded in a nickel boat for analysis. Hg was measured in RBC instead of whole blood as the samples had been centrifuged to obtain plasma for another study and plasma is not a recommended matrix to analyse Hg (see Espín et al. 2021), as most MeHg is bound to erythrocytes. In addition, analysis of total Hg in RBC is considered a suitable proxy of recent MeHg exposure (Berglund et al. 2005). Hence, RBC samples from NG were loaded in nickel boats (ca. 0.1 g, wet weight; ww) and analysed using the same technique. The calibration curve was traced with ten points in duplicate 0–1004 ng). The method was tested using certified reference material (CRM) (Hg Standard for AAS, Fluka, 1000 mg L^−1^ Hg in 12% nitric acid, prepared with high purity Hg metal, HNO^3^- Trace SELECT® and water Trace SELECT® Ultra) (mean recovery of 5 replicates 104.2% and coefficient of variation for repeatability 11.4%). The limit of detection was 0.005 ng. Blanks were included every 10 samples. Periodically, certified reference material (CRM; TORT-2, lobster hepatopancreas, National Research Council Canada) was analysed in duplicates for testing precision and accuracy of the method, obtaining a recovery percentage of total Hg of 108.14% ± *4.1*% (mean ± standard deviation) and repeatability 3.7% from 7 replicates of CRM diluted to 1 ppm. To compare Hg in blood with other studies, values in RBCs were transformed using average values of haematocrit in NG nestlings from Norway (41.39%, Briels et al., unpublished data) and density of 1.1 g mL^−1^ for blood pellets (Ortiz-Santaliestra et al. 2015).

Feather barbs were washed with ultrapure water, dried at room temperature, cut with scissors (< 1 mm pieces) and loaded into tin cups for analysis of stable carbon (*δ*^13^C) and nitrogen (*δ*^15^N) isotopes at the Laboratory of Oceanology at the University of Liège (Belgium), using continuous flow elemental analysis isotope ratio mass spectrometry with a Vario MICRO cube elemental analyser (Elementar Analysensysteme GmBH, Hanau, Germany) coupled to an IsoPrime100 mass spectrometer (Isoprime, Cheadle, United Kingdom). Sucrose (IAEA-C6, *δ*^13^C, mean ± *SD* =  − 10.8 ± *0.5*‰) and ammonium sulphate (IAEA-N2, *δ*^15^N mean ± *SD* =  + 20.3 ± *0.2*‰) were used as CRM and were calibrated against the international isotopic references. Standard deviations on multi-batch replicate measurements of lab standards (fish tissues) analysed interspersed among the samples (2 lab standards for 15 samples) were 0.1 and 0.3 ‰ for *δ*^13^C and *δ*^15^N.

To test differences between species in feather Hg, we used linear mixed models (LMMs; Bolker 2015). To avoid pseudoreplication, all models included *nest* as a random factor. To assess whether the model containing species performed better in predicting feather Hg concentrations, we compared the log likelihood with the null model using ANOVA (Burnham and Anderson, 2002). Correlations between the variables (*δ*^13^C and *δ*^15^N in feathers, Hg in feathers and in RBCs from NG) were assessed using Spearman (*Rho*) correlation tests. LMMs were also used to assess the relationship between *δ*^13^C and *δ*^15^N isotopes and Hg in feathers, again including *nest* as a random factor and comparing the log likelihood with the null model using ANOVA. If the species model performed significantly better than the null model, we ran the models separately by species, but if not, they were run together in a single model including *species* as a factor. We analysed both univariate models and models that included the two isotopes together. Before including the two isotopes in the model, the variance influence factor (VIF) was checked for multicollinearity between isotopes (Dormann et al. 2013). Models were run only with two isotopes if found VIF < 6. In all cases, the log-likelihood was compared with the null model using ANOVA. All data analyses were performed using the R statistical software (R Core team 202 0) and SPSS v. 25. All tests were two-tailed, and the level of significance was set at *α* = 0.05. Values were shown as mean ± *SD* (median; range) and estimates from the LMMs were shown as mean and 95% confidence intervals.

## Results

**Fig. 1:**
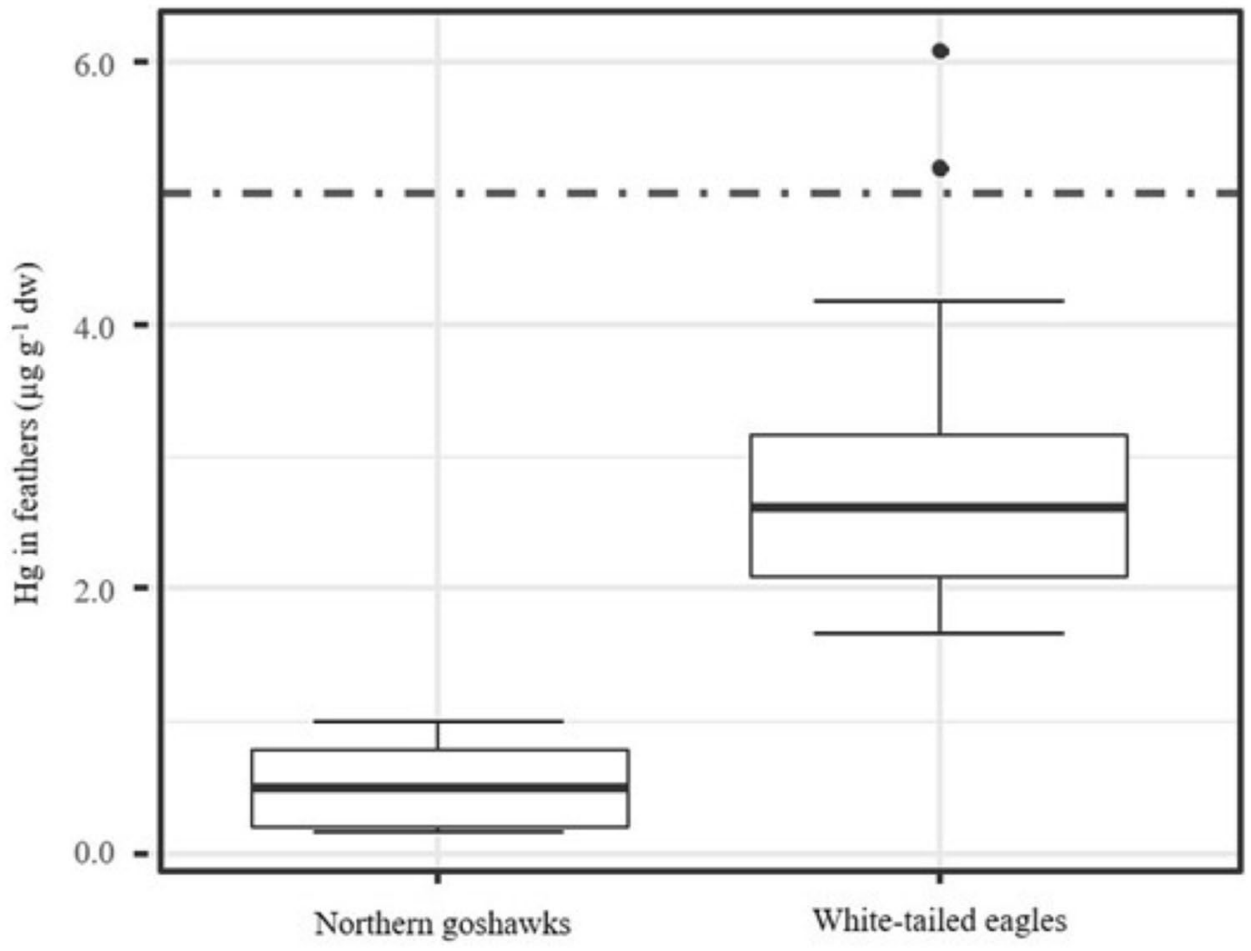
Hg concentrations (µg g^−1^, dw) in body feathers of Northern goshawk and white-tailed eagle nestlings from Norway. The boxplot represents mean (midline), standard error (SE, box), standard deviation (SD, whiskers) and outliers (dots). The dashed line indicates the threshold for sublethal effects of 5.00 µg g^−^.^1^ dw Hg feathers (Eisler 1987; Burger and Gochfeld 1997)

Hg was found in all the feather samples and concentrations (dry weight) were 0.51 ± *0.34* (0.50; 0.16–1.01) µg g^−1^ in NG and 3.01 ± *1.34* (2.62; 1.66–6.08) µg g^−1^ in WTE. The LMM showed that the *species* factor was significant in explaining the Hg concentration in feathers (LMM;* p* < 0.001, estimate (WTE) = 2.51, 95% CI = 1.26, 3.76). WTE had higher feather concentrations than NG (Fig. 1). RBC Hg concentrations in NG were 0.03 ± *0.01* (0.03; 0.02–0.05) µg g^−1^ ww. LMM found no relationship for Hg concentration in the feather with RBC Hg (*log-likelihood* = 3.23, *p* = 0.28, estimate =  − 0.01; 95% CI =  − 0.02, 0.00).

**Fig. 2:**
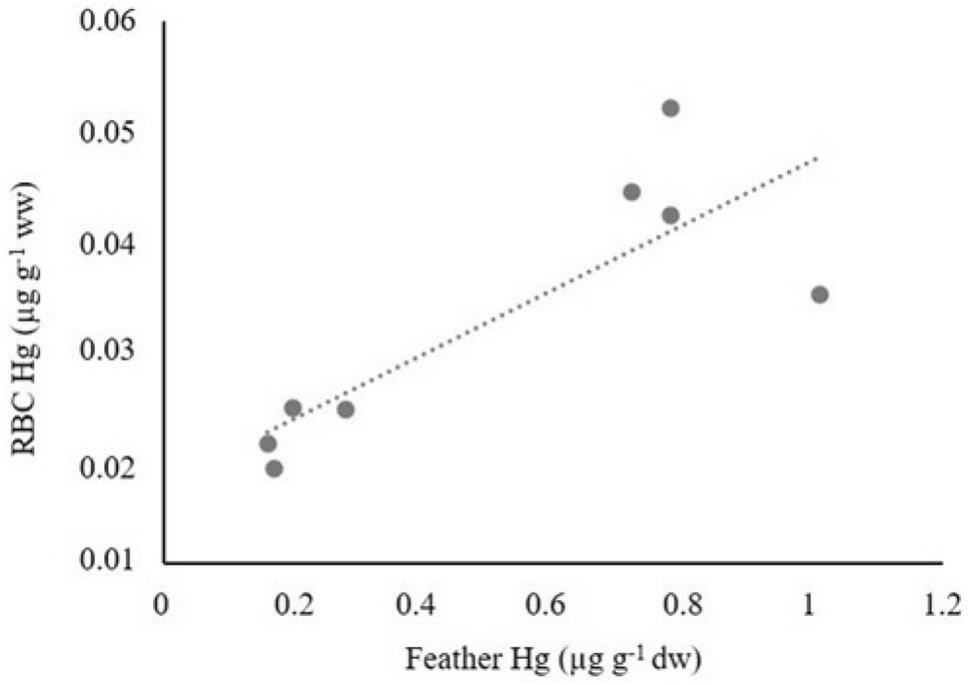
Correlations between Hg concentrations in feathers and red blood cells (RBC) of NG (*Rho* = 0.77, *p* = 0.03)

Hg concentrations in RBCs of NG nestlings were positively correlated with feather Hg concentrations (*Rho* = 0.77, *p* = 0.03; Fig. 2).

**Fig. 3:**
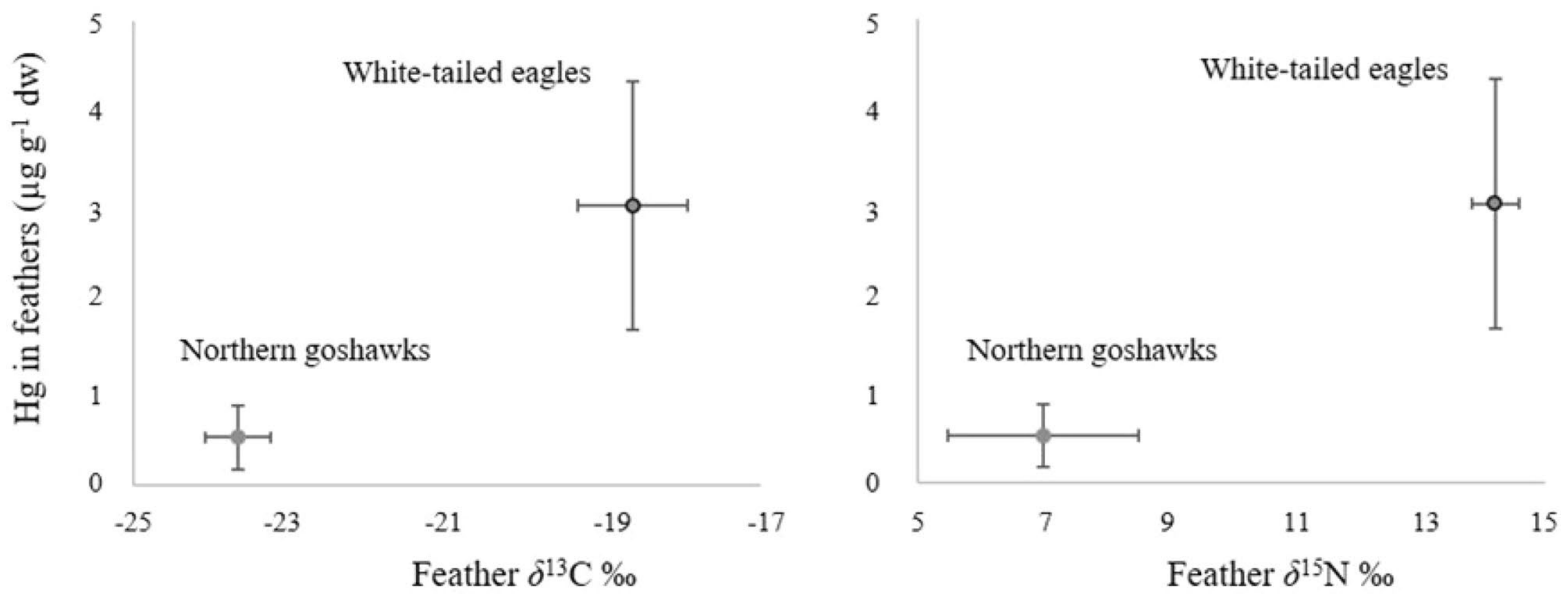
Mean and *SD* of *δ*^13^C, *δ*^15^N and Hg in nestling body feathers of white-tailed eagle and Northern goshawk nestlings

As previously reported (Gómez-Ramírez et al. 2017), SIs values in the feathers differed between the species, with *δ*^13^C and *δ*^15^N body feather values higher in WTE than in NG (Fig. 3). Values of SIs per sampled species in relation to feather Hg concentrations are represented in Fig. 3.

Linear mixed models showed no significant relationship between SIs and Hg in the NG nestling body feathers (LMM* δ*^13^C, log-likelihood = 2.83, *p* = 0.55, estimate = 0.06, 95% CI =  − 0.18, 0.31; LMM* δ*^15^N*,* log-likelihood = 3.12, *p* = 0.33*,* estimate = 0.04, 95% CI =  − 0.07, 0.16), nor for WTE nestling body feathers (LMM *δ*^13^C, log-likelihood =  − 21.74, *p* = 0.24, estimate =  − 0.59, 95% CI =  − 1.65, 0.47; LMM *δ*^15^N, log-likelihood =  − 20.44, *p* = 0.107, estimate = 1.56, 95% CI =  − 0.22, 3.36). The models including the two isotopes were not shown to perform significantly better than the null model in NG (LMM*, l*og-likelihood = 3.12, *p* = 0.24), but were nearly significant for WTE (LMM, log-likelihood =  − 19.16, *p* = 0.07). However, when Spearman correlations between SIs and Hg were tested, these were positive and significant for both *δ*^13^C (*Rho* = 0.69, *p* = 0.047) and *δ*^15^N (*Rho* = 0.71, *p* = 0.037) in the NG nestling body feathers, but not so in the WTE (*δ*^13^C *Rho* =  − 0.19, *p* = 0.51; *δ*^15^N *Rho* = 0.39, *p* = 0.19).

## Discussion

Blood Hg resulted as 0.015 ± *0.005* (0.014; 0.009–0.024) µg mL^−1^ ww. These levels were very similar to NG nestlings born in 2015 in the same study area and in a non-polluted area in South-eastern Spain but much lower than in nestlings from a more urbanised and agricultural area in central Norway (Dolan et al. 2017, Table 1).

**Table 1:** Concentrations of Hg found in nestling raptors in blood (RBC) and feathers from this and other studies

Species	Matrix	Concentrations (median)	Location	References
*Accipiter nisus*	Blood	*0.014 µg mL*^−1^* ww*	*Northern Norway*	*This study*
		0.014 µg mL^−1^ ww	Northern Norway	Dolan et al. (2017)
		0.012 µg mL^−1^ ww	South-eastern Spain	
		0.028 µg mL^−1^ ww	Central Norway	
	Feathers	*0.50 µg g*^−*1*^* dw*	*Northern Norway*	*This study*
		0.83 µg g^−1^ dw (geometric mean)	Minnesota, USA	Keyel et al. (2020)
		0.16 µg g^−1^ dw	Northern Norway	Dolan et al. (2017)
		0.18 µg g^−1^ dw	South-eastern Spain	
		0.31 µg g^−1^ dw	Central Norway	
		2 µg g^−1^ dw	Finland	Solonen and Lodenius (1990)
*Haliaeetus albicilla*		*2.62 µg g*^−*1*^* dw*	*Northern Norway*	*This study*
*Falco peregrinus*		0.67 µg g^−1^ dw (mean)	Southern Nevada, USA	Barnes and Gerstenberger 2015
		5.82	Lake Mead National Recreation Area, USA	
*Strix aluco*		0.74 µg g^−1^	Central Norway	Bustnes et al. unpublished
*Bubo bubo*		0.24 µg g^−1^ dw	Southern Spain	Espín et al. (2014a)
		0.7 µg g^−1^ dw	Finland	Solonen and Lodenius (1990)
*Strix aluco*		0.4 µg g^−1^ dw		
*Strix uralensis*		0.4 µg g^−1^ dw		
*Strix nebulosa*		0.6 µg g^−1^ dw		
*Asio otus*		0.2 µg g^−1^ dw		
*Aegolius funereus*		0.4 µg g^−1^ dw		
*Pandion haliaetus*		4 µg g^−1^ dw		
		2.76 µg g^−1^ dw	Delaware Bay, USA	Rattner et al. (2008)
		3.9 µg g^−1^ dw (mean)	Western Canada	Guigueno et al. (2012)

Hg in feathers of NG were in the range of nestlings of the same species in Norway and Spain (Dolan et al. 2017), but also in other terrestrial diurnal birds of prey of similar diets from USA, Spain and different owl species from Finland and Spain (see Table 1), but lower than the Finnish NG (Solonen and Lodenius 1990) or Norway (see Table 1). In WTE, similar concentrations were found in nestling feathers of osprey (*Pandion haliaetus*) from different places in USA, Canada and Finland (see Table 1) and in peregrine falcons fledglings feeding on aquatic birds from Lake Mead National Recreation Area in USA (Barnes and Gerstenberger 2015).

In our study, only one feather per individual could be analysed, as the rest were used for other analyses (Gómez-Ramírez et al. 2017). There seems to be some controversy in this regard, as according to some authors (Peterson et al. 2019), to avoid intra-individual variations, several grown feathers should be used, while other studies reported very small differences among individual feathers (Roque et al. 2016; Ekblad et al. 2021). Nevertheless, the positive and significant relationship between feather and blood concentrations of Hg in NG, also found in other studies on nestlings (Dolan et al. 2017; Espín et al. 2014a), supports the usefulness of body feathers to biomonitor and estimate Hg exposure.

Emissions of Hg in Norway have been significantly reduced since 2007, due to restrictions and improvements in waste treatments (Climate and Pollution Agency 2010). However, this metal was quantified in all the samples analysed. This is in line with its well-known ubiquity and long range transport (UNEP 2013) and the bioaccumulation and biomagnification potential of MeHg, through the food chain. WTE feathers from adult birds showed a decrease in Hg from the 1960s to 2015 in the Norwegian population (Sun et al. 2019). Similarly, a trend study in moss between 1995–2000 showed a general decline in the whole country (Harmens et al. 2008). On the contrary, feathers of female tawny owls from central Norway showed no temporal trend between 1986–2005 (Bustnes et al. 2013). Although our results only provide a snapshot on exposure in 2014, these can be used as a baseline in future studies to assess temporal trends.

SIs have been used to relate contaminant exposure to dietary sources (Inger and Bearhop 2008). However, its utility in feathers has been discussed, mainly based on the statement that contaminants (especially Hg) and SIs are integrated into feathers over different time periods, which results in spurious relationships with no biological meaning (Bond 2010). However, unlike in adult feathers, this utility seems to be clear in the case of body feathers from nestlings, as strong correlations are usually found in different species including seabirds, ardeids and eagles (Rodríguez et al. 2013; Einoder et al. 2018; Cherel et al. 2018; Badry et al. 2019). In agreement with similar studies on WTE and NG from the same study areas (Eulaers et al. 2013; Løseth et al. 2019) as well as general expectations (Inger and Bearhop 2008), the SIs results show the dependence on two different ecosystems of the two investigated species. While NG *δ*^13^C values fall within the expected range for terrestrial birds (mean ± *SD*: − 22.9% ± *2.6*%), those for WTE are as to be expected for a carnivorous predominantly marine species (mean ± *SD*: − 18.8 % ± *2.2*%), (Kelly 2000), and in fact two different stable isotope niches that do not overlap (Gómez-Ramírez et al. 2017). This differentiation in dietary ecology is reflected in significant differences in Hg concentrations between species and echoes earlier observations of the marine environment presenting higher contaminant accumulation (Gómez-Ramírez et al. 2017). However, correlations between Hg and *δ*^13^C followed different patterns in each species. The positive correlations in both feather and blood from NG suggest marine dietary input. The negative correlation in WTE is suggested to be related to freshwater or terrestrial food sources (Boutton 1991). In this case, the proximity to coast and therefore, to anthropogenic sources may be the cause of higher levels, as already suggested for perfluorinated flame retardants (Gómez-Ramírez et al. 2017).

The positive correlations between Hg and *δ*^15^N values are indicative of biomagnification through the food chain, as already reported in both terrestrial and marine species (Bearhop et al. 2000; Eulaers et al. 2013). In fact, the samples with the highest concentrations (6.08 and 5.19 µg g^−1^ dw,), belonging to WTE, also showed high *δ*^15^N (14.43 and 14.79%, respectively). Despite this, and probably due to the small sample size, we found no significant relationship between SIs and Hg in feathers. The lack of significance can also be due to a low variability of trophic levels in their prey items (Góngora et al. 2018). Nevertheless, the trophic level of both species could not be compared because prey items were not analysed.

Field and laboratory studies have shown alterations after exposure to Hg in birds, such as hepatotoxicity, immunotoxicity, endocrine disruption and developmental toxicity, including suppression of baseline corticosterone (Herring et al. 2012). The threshold level for sub-lethal effects has been suggested at 5 µg g^−1^ dw Hg in feathers (Eisler 1987; Burger and Gochfeld 1997). In contrast, Sun et al. (2019), based on reproductive and toxicity symptoms, proposed a safe level in WTE at 40 µg g^−1^ dw in feathers, well above any of the concentrations found in the current study. Only two WTE from different nests showed feather Hg concentrations just above the 5 µg g^−1^ dw threshold (6.08 and 5.19 µg g^−1^ dw, see Fig. 1). However, studies in eagle owl nestlings suggested that lower Hg feather concentrations (0.32 µg g^−1^ dw, close to our results in NG feathers) may alter antioxidant enzymes and increase lipid peroxidation in RBCs (Espín et al. 2014a, b). Although effects at the biochemical level in these individuals cannot be excluded without further investigation of sub-lethal effects, the estimated blood Hg concentrations are below 0.03 µg mL^−1^ ww, the level associated to increased concentrations of superoxide dismutase and thiobarbituric acid reactive substances in eagle owls (Espín et al. 2014a, b). However, susceptibility to Hg is very variable within and among species, and the toxic levels vary for different endpoints (e.g. hatching, deformities, cognitive effects). For further risk assessment, increased sample sizes and analysis of selenium (Se) in feathers is recommended, as the interaction of both metals has been related to a decreased Hg toxicity. The assessment of Se:Hg molar ratios may provide additional information about the potential toxicity of Hg (Burger et al. 2013).

Concentrations of Hg are within the range of background contamination levels for birds. According to the SIs analyses, variation and inter-specific differences in Hg levels seem to be related to dietary plasticity. Although the concentrations of Hg are mostly below the suggested safe levels for other birds of prey, further studies regarding sub-lethal effects with larger sample sizes and including the ratio Se–Hg are recommended (Burger et al. 2013). This study provides baseline data to be compared with future biomonitoring studies using blood and feathers from birds of prey in Northern Norway.
